# Colonial Medical Research

**Published:** 1954-07

**Authors:** H. S. L. Heller

**Affiliations:** Professor of Pharmacology, University of Bristol


### COLONIAL MEDICAL RESEARCH

BY

H. S. L. HELLER, M.D., Ph.D., M.R.C.P.
*Professor of Pharmacology, University of Bristol*

Most medical practitioners in this country know little about the research sponsored by the Colonial Office. Some of this work has distinct bearings on medical and economic problems of countries in the temperate zone. Therefore it may not be out of place to say a few words about this aspect of British Colonial stewardship.

The central organizing body is the Colonial Medical Research Committee (C.M.R.C.). It is aided by regional organizations in some of the Colonial territories, in East Africa by the East African Bureau of Research in Medicine and Hygiene and the East African Standing Advisory Committee for Medical Research, and in West Africa by a similar Standing Advisory Committee which will soon be enlarged to a West African Council for Medical Research.

The C.M.R.C. administers funds allocated to it under the 1945 and 1950 Colonial Development and Welfare Acts. Up to 1953 £12,200,000 has been provided, 14.2 per cent of which has been allotted to medical research directly, and a further 27.1 per cent to work closely linked with medical problems like tsetse and trypanomiasis research. The way in which these monies are used varies widely: research units are maintained in the Colonies, assistance is given to research programmes undertaken by the Medical Departments of Colonial territories, the training of young medical workers is subsidized, special grants are made to research institutions or university departments in the United Kingdom working on problems of interest to the Colonies. A new development which the C.M.R.C. seeks increasingly to foster is the provision in Colonial centres of facilities for senior research workers from British universities for the short-term study of specific problems.

The forms of research work done in the Colonies include medical surveys, clinicopathological studies and therapeutic trials; infectious and parasitic diseases, nutritional deficiency and endocrine diseases, and physiological problems are studied.

The physiological research is by no means an academic extravagance; it is necessary to provide a basis for clinical work. Thus one of the difficulties of investigating a pathological condition in an African territory is the lack of normal or control data. Thanks to the labour of several generations of physiologists and clinical pathologists, a standard text-book usually contains reliable ranges for, say, the level of most blood or plasma constituents in Europeans or North Americans of most age-groups. Similarly there are numerous investigations giving the results of function tests on various organs of healthy white people. However, very few such facts are known about the inhabitants of Colonial territories. It is, of course, not to be assumed *a priori* that differences will be found, and, if found, that they are due to different racial characteristics. They may be due to the different climatic conditions or the local diets. But whether such differences can be found or not, it is clear that the investigator is not entitled to interpret findings in any pathological condition without data on a representative sample of healthy subjects of the same population. To give an example: a small group of workers from the Department of Pharmacology, Bristol, recently went out to Kampala, Uganda, to collect data on the water metabolism and the renal function of infants and adults suffering from nutritional oedema and liver disease. When we came to apply simple renal function tests like measurements of the ability of the kidneys to dilute or concentrate the urine, we realized that there was no information on Africans in Uganda or any other part of that continent. Similarly, there have been very few estimations of plasma sodium and potassium levels in Africans. Therefore healthy infants and adults had first to be investigated. But this was by no means easy. There were curious difficulties. For although Africans are not averse to injections (in fact they like them and expect much from them), the *removal* of body fluids is a different matter. This is frequently resented, probably because the African fears that the material will be used for magical purposes. However, when matters are carefully explained to them, it is usually possible to obtain the consent of the African patient or the patient's mother to remove small samples of blood. Assuming then that a number of volunteers have been assembled, will the results obtained be acceptable? Will they really yield "normal" values? It must be remembered that the incidence of parasitic or infectious diseases in many tropical areas is very high even amongst subjects who are apparently well enough. In Uganda, for instance, malaria, hook-worm infestation and amoebiasis have to be thought of. Moreover, nutritional deficiencies are widespread. Gross cases will be admitted to hospital but the majority of the "controls" available may be suffering from the same condition to a minor degree. A statement of Davies and Trowell (cited from Brock and Autret) bears this out. They state that in Kampala there is hardly an adult African liver which does not show fibrosis to some degree, at least when examined microscopically. Such difficulties as these lead to a better appreciation of the work of the pioneer physiologists, done at centres like Makerere Medical College, Kampala, Uganda, the Hot Climate Physiology Research Unit at Oshodi, Nigeria, the M.R.C. Laboratories at Fajara, Gambia and others.

Besides this physiological and biochemical research there are epidemiological and nutritional surveys which will mould future clinical work. The East African Medical Survey not only studies the incidence of diseases like tuberculosis, malaria, relapsing fever and schistosomiasis but also chooses areas for experimental vaccination and for therapeutic trials. Two other examples of recent surveys are an investigation of the epidemiology of human trypanosomiasis in the Gambia, in the course of which over 55,000 persons were examined, and an inquiry into the incidence of endemic goitre in Nigeria. Such surveys may have their peculiar difficulties. For instance, when the victims of filariasis are asked to come to the hospital at night they are reluctant to do so because lions and other wild beasts make their journey hazardous. Nor is the vampire-like activity of research workers who wish to visit infested villages to make blood-films at night particularly appreciated.

There are closely related projects designed to identify potential insect and other vectors of diseases, to find their habitat and breeding habits and to learn about seasonal incidence. Much of this work is done in collaboration with entomologists. Astonishing results (astonishing at least to a medical man who knows little about tropical diseases) are sometimes obtained: it was found in Borneo, for instance, where malaria is hyper-endemic, that the previously accepted vector *Anopheles maculatus* is almost certainly harmless, and that the chief vector throughout the land is another mosquito, *A. leucosphyrus*, which is not "house-haunting" but breeds in jungle-covered hill ravines. Conventional anti-mosquito measures like the oiling of pools, spraying of buildings with D.D.T. and drainage will therefore not prevent Borneo malaria, but clearance of scrub, sufficient to admit sunlight, results in quick eradication of *A. leucosphyrus* breeding. It would take too long to mention similar research programmes in any detail. Mere quotation of some subjects and sites of field work may be sufficient to show the variety and wide territorial spread of inquiries in progress:

*East Africa:* distribution of mosquitoes transmitting filariasis; resting habits and physiology of malaria mosquitoes; relapsing fever research (Kikuyu Reserve, Kenya, and Usambara highlands, Tanganyika). Study of snails on Ukara Island in a search of schistosoma species; isolation of polyomyelitis virus (by the Virus Research Institute, Entebbe).

*West Africa:* study of transmission of filarial infection in the rain-forests and grass-lands of the British Cameroons; interrelation between helminthic infestation and nutrition (Gambia); the distribution of transmitters of the guinea-worm (Nigeria); malaria in pregnancy; study of yellow fever epidemics and isolation of strains (Virus Research Institute, Lagos).

*Malaya:* study of vectors of filarial infections: malaria control in rural areas; investigation of the animal reservoirs and vectors of yellow fever, dengue, encephalitis and leptospirosis (by the Colonial Office Scrub-typhus Unit in collaboration with a unit from the U.S. Army Medical Service).

*Trinidad:* entomological research on malaria mosquitoes and the effect of D.D.T. and gammexane. Investigations such as these are frequently combined with therapeutical trials which are very necessary since well-known and accepted drugs may have to be tried on newly discovered strains or newly discovered drugs may have to be tested.

While much time and money is spent on " tropical " infections, increasing attention is being paid to the study of nutritional deficiency conditions. The prevalence of these diseases in many tropical countries has only been recognized in recent times. This is to some degree a result of medical experience in European countries during and after the two great wars. Deficiencies of subsidiary food factors, i.e. vitamins, could be studied in peace-time, but it is mainly under war-time conditions that gross under-nutrition and lack of one or more of the chief food constituents (proteins, fats) occur. These circumstances led to the intensive study and better understanding of starvation oedema and nutritional liver damage, and to the recognition of a peculiar syndrome in infants fed on diets poor in proteins but rich in carbohydrates, termed " Mehlnaehrschaeden " by German investigators. Similar conditions known as " Kwashiorkor " were subsequently reported from East and South Africa. Depigmentation of skin and hair, and dermatoses seem to be more prominent in the tropics than in Europe. However, when, at a recent occasion, the opportunity arose to compare African infants with kwashiorkor with Italian children of the same age-group suffering from nutritional protein deficiency, little doubt was left in the writer's mind about the essential identity of the two conditions. Lack of protein is now generally agreed to be the main cause of this syndrome although regional differences in the symptomology make it likely that additional factors are involved: the amino-acid composition of the protein in the plants used as staple food in various regions may differ, there may be subsidiary deficiencies of one or several vitamins, toxic substances may be contained in the vegetable diets fed, or there may be differences in the proportions of the main food ingredients (carbohydrate/protein ratio). The clinical picture remains nevertheless sufficiently clear to have led to the recognition of kwashiorkor in areas as widely dispersed as East and West Africa, the Belgian Congo, South Africa, the Philippines, the West Indies and Mexico.

It seems likely that adult forms of kwashiorkor exist, both in an acute form frequently seen in Southern India (which according to a personal communication from Dr. C. Gopalan of the Nutritional Laboratories at Koonoor closely resembles kwashiorkor in weanling infants) and in a chronic form seen by us in Uganda in young adults (these patients are usually admitted with liver cirrhosis, oedema and ascites.

During six weeks at the Mulago Hospital, Kampala, we saw twenty infants suffering from severe kwashiorkor. The high incidence and fatality of this disease raises major problems of prevention and therapy in Uganda and many other tropical countries. Both problems are complicated. The main therapeutic task would appear to consist in administering sufficient amounts of proteins yielding mixtures of amino-acids of high biological value. Milk seems the simplest answer, but it has not proved to be entirely satisfactory. Moreover, the amounts of milk needed would be very considerable and in East Africa for instance—where the milk yield of the native cattle is very poor—such treatment would be too costly. A special M.R.C. unit is being organized in Kampala (with the help of the Colonial M.R.C. and the Government of Uganda), to study, amongst other nutritional problems, the use of more easily available plant proteins in the treatment and in the prevention of kwashiorkor. However, measures other than dietary may have to be used for treatment, since a number of children die in spite of prompt protein administration. More rapid reduction of the oedema may be necessary in some patients; but such treatment presupposes greater knowledge of the causal factors, and of renal function in this disease. It was with this aspect of kwashiorkor that the group of workers from Bristol University were concerned.

Prevention of kwashiorkor poses, if anything, an even more difficult problem than its therapy. Firstly, a very large number of people has to be protected; secondly, purchase of the right type of food is usually beyond the means of an African family; thirdly, the food habits of some tribes would have to be changed. The problem of combatting kwashiorkor is therefore not only medical, it is economic and sociological as well.

Sufficient has been said to convey an impression of the scope and diversity of medical research in British Colonial territories; and if, as many believe, world peace and security depend on the improvement of living conditions in the most needful areas, the general importance of much of this work will be easily recognized.

I am much indebted to Dr. R. Lewthwaite, O.B.E., F.R.C.P., Director of Medical Research, Colonial Office, for reading this article before its publication.

